# Dose-Independent Therapeutic Benefit of Bone Marrow Stem Cell Transplantation after MI in Mice

**DOI:** 10.3390/biomedicines8060157

**Published:** 2020-06-11

**Authors:** Nicole Zarniko, Anna Skorska, Gustav Steinhoff, Robert David, Ralf Gaebel

**Affiliations:** 1 Department of Cardiac Surgery, Rostock University Medical Center, 18059 Rostock, Germany; nicole.goedecke@charite.de (N.Z.); anna.skorska@med.uni-rostock.de (A.S.); gustav.steinhoff@med.uni-rostock.de (G.S.); ralf.gaebel@med.uni-rostock.de (R.G.); 2 Department Life, Light & Matter (LL&M), University of Rostock, A.-Einstein-Str. 25, 18057 Rostock, Germany

**Keywords:** myocardial infarction, stem cells, autologous cell therapy, pre-clinical, scientific advance

## Abstract

Several cell populations derived from bone marrow (BM) have been shown to possess cardiac regenerative potential. Among these are freshly isolated CD133^+^ hematopoietic as well as culture-expanded mesenchymal stem cells. Alternatively, by purifying CD271^+^ cells from BM, mesenchymal progenitors can be enriched without an ex vivo cultivation. With regard to the limited available number of freshly isolated BM-derived stem cells, the effect of the dosage on the therapeutic efficiency is of particular interest. Therefore, in the present pre-clinical study, we investigated human BM-derived CD133^+^ and CD271^+^ stem cells for their cardiac regenerative potential three weeks post-myocardial infarction (MI) in a dose-dependent manner. The improvement of the hemodynamic function as well as cardiac remodeling showed no therapeutic difference after the transplantation of both 100,000 and 500,000 stem cells. Therefore, beneficial stem cell transplantation post-MI is widely independent of the cell dose and detrimental stem cell amplification in vitro can likely be avoided.

## 1. Introduction

The treatment with stem or progenitor cells offers a potential chance of true regeneration and regrowth of irreversibly damaged tissue as well as anti-inflammatory effects. Intramyocardial stem cell transplantation has been investigated as a novel therapy option since Asahara and coworkers first found evidence of bone marrow (BM) cell participation in cardiac healing [1]. In vivo studies, as well as clinical trials, have been conducted using widely mixed cell populations such as total nucleated or mononuclear BM-derived cells as well as purified cells of specific cell types [2,3,4]. However, no single most effective cell type has been identified yet. Hematopoietic stem cells (HSC) as well as mesenchymal stem cells (MSC) have been studied in the most detail [2,5]. CD133^+^ cells, representing highly immature HSC with hemangioblast potential, have been shown to increase cardiac regeneration in animal studies as well as phase I/II clinical trials [6,7,8]. Hematopoietic cells are physiologically among the first cell types influenced by MSC-based paracrine activity [9]. Mechanisms of action currently discussed include paracrine effects of transplanted cells as well as differentiation of stem/progenitor cells in situ [10]. In particular, MSC show a pronounced secretion of growth factors such as vascular endothelial growth factor (VEGF) and basic fibroblast growth factor (bFGF) [11,12] and pro-inflammatory as well as anti-inflammatory mediators such as IL-6, IL-8 or IL-10 [13,14,15]. VEGF has been linked to the myocardial regenerative potential of these cells by Zisa and colleagues [16]. However, MSC are usually isolated relying on their plastic adherence and expanded for several weeks ex vivo before application, and may carry an enhanced risk of uncontrolled cell division, leading to malignancies due to the unphysiological environment they are confronted with [17,18]. Meanwhile, those possible pitfalls seem to be solved by the involvement of the Product Quality Review (PQR) using validated testing assays (cell identity morphology, sterility, karyotype, and efficacy tests) at certain passages before clinical use [19,20]. Nevertheless, variations in culture conditions heavily led to varying findings regarding MSC behavior and features in vitro [21,22]. Based on this observation, there is a need for alternative selection methods. Hence, CD271 or low affinity nerve growth factor receptor (LNGFR) has been shown to identify a population within BM which contains all MSC [23,24]. In the present work, we made use of the fact that a prospective selection of MSC relying on CD271^+^-based immunomagnetic separation is possible, thereby avoiding culture expansion. However, these stem cells are rare and the total number of CD271^+^ cells—as of any other stem cell population—that may be obtained from BM is very limited. Although the optimal cell dosage for therapy has not been determined yet, it is plausible that the number of transplanted cells may be a major limiting factor. Therefore, we here analyzed a potential dose dependency of human BM-derived CD133^+^ as well as CD271^+^ stem cells for their cardiac regenerative properties after myocardial infarction (MI) in a small animal setting. 

## 2. Materials and Methods 

### 2.1. Bone Marrow Aspiration

Bone marrow (BM) was aspirated from informed donors who gave written consent regarding the use of their BM for research according to the Declaration of Helsinki. The ethical committee of the University of Rostock approved the presented study (registered as no. A 2010 23) on 29 April 2010. BM samples were obtained by sternal aspiration from patients undergoing coronary artery bypass graft surgery at Rostock University Hospital, Rostock, Germany. Anticoagulation was achieved by heparinization with 250 i.E./mL sodium heparine (B. Braun Melsungen GmbH, Melsungen, Germany). 

### 2.2. Cell Isolation

Mononuclear cells from all samples were isolated by density gradient centrifugation on 1077 Lymphocyte Separation Medium (LSM; PAA, Pasching, Austria). CD133^+^ and CD271^+^ cells were enriched by positive magnetic selection using the magnet activated cell sorting (MACS) system (Miltenyi Biotec, Bergisch Gladbach, Germany), using direct labeling in case of CD133^+^ and an indirect labeling (CD271-APC/anti-APC-micro beads; Miltenyi Biotec) for CD271^+^ cells. Purity and viability of all cell isolations were verified by flow cytometry as previously described [24,25,26]. 

### 2.3. Experimental Design of the Animal Model 

The federal animal care committee of LALLF Mecklenburg-Vorpommern (Germany) approved the study protocol (approval number LALLF M-V/TSD/7221.3-1.1-088/11). Female, nine to twelve-weeks old severe combined immunodeficiency (SCID) beige mice (strain CB17.Cg-*Prkdc^scid^Lyst^bg-J^*/Crl) were purchased from Charles River Laboratories (Sulzfeld, Germany) and randomly assigned to 6 experimental groups: MI, given as low-dose application with 100,000 implanted human CD133^+^ (MI133L) or CD271^+^ (MI271L) stem cells, or given as high-dose application with 500,000 cells (MI133H; MI271H). Untreated MI animals (MIC) and Sham operation (SHAM) served as controls. 

### 2.4. Generation of Reperfused MI in Mice and Stem Cell Implantation 

Mice were anesthetized with Pentobarbital (50 mg/kg, intraperitoneal; Narcoren^®^, Boehringer, Ingelheim, Germany). After thoracotomy and preparation, the left anterior descending coronary artery (LAD) was ligated. After 45 min each mouse received an intramyocardial injection of MACS^®^ buffer (10 µL; Miltenyi Biotec) mixed with 10 µL of BD Matrigel^TM^ Matrix (BD Biosciences, San Jose, CA, USA). For cell application 100,000 (low dose, L) or 500,000 (high dose, H) stem cells of the respective source suspended in 10 µL of MACS buffer were mixed with 10 µL of BD Matrigel^TM^ Matrix. Injections of 4 × 5 µL were given along the border of blanched myocardium and ligation was removed. SHAM operated mice underwent identical surgical procedures without LAD-ligation but followed by intramyocardial MACS^®^ buffer/BD Matrigel^TM^ Matrix injection without cells. 

### 2.5. Left Ventricular Catheterization and Heart Tissue Analyses

The analysis of cardiac functions, organ harvesting, fibrosis analysis, determination of blood vessels and RNA isolation as well as polymerase chain reaction was described by us previously [24]. 

### 2.6. Statistical Analysis

The calculated standard deviation of the mean value (Mean ± SD) is given. The statistical evaluation was carried out using SPSS Statistics (Version 22, IBM, Armonk, NY, USA). A *p*-value < 0.05 was assumed to be statistically significant. The normal distribution test was carried out using the Shapiro–Wilk test. For a given normal distribution, significant differences between the mean values of the groups were determined using paired *t*-tests, otherwise using paired Mann–Whitney *U* tests. The Kaplan–Meier survival rates were compared using a log-rank test. 

## 3. Results

### 3.1. Survival Rate

**Figure 1:**
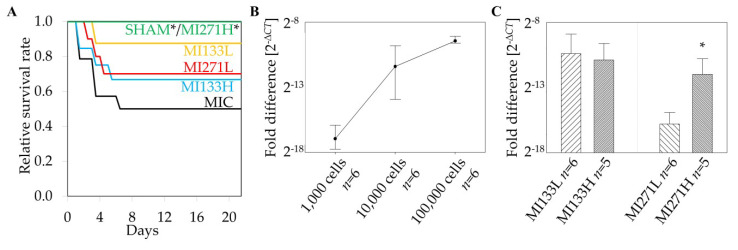
Therapy-associated relative survival rates and stem cell retention. Significant survival rate for SHAM operated as well as high-dose CD271^+^ stem cell treated mice (MI271H) compared to untreated myocardial infarction (MIC); * *p* = 0.03 vs. MIC (log-rank test; (**A**)). Detection threshold of 1000 cells using human GAPDH [24] (**B**). No significant difference between low and high-dose CD133^+^ stem cell treatment in contrast to the MI271L/MI271H group 3 weeks post MI in mice; Mean ± SD; * *p* ≤ 0.05 vs. MI271L (Mann–Whitney U test; (**C**)).

In order to evaluate their cardiac regenerative potential, 100,000 (low dose) or 500,000 (high dose) stem cells were transplanted into SCID beige mice after cardiac ischemia/reperfusion induced by short-time ligation of the left anterior descending artery. While high-dose CD271^+^ stem cell treated mice (MI271H; *p* = 0.03) revealed a significantly better survival rate than the untreated MI (MIC) group, there was a clear trend towards better survival in the therapy groups MI133H, MI133L, and MI271L compared to the MIC controls (Figure 1A). The survival rates 21 days after the onset of the experiment were 100% in the SHAM group (*n* = 7), 50% in the MIC group (*n* = 14), 88% in the MI133L group (*n* = 8) and 70% in the group MI271L (*n* = 10), 67% in the MI133H group (*n* = 12) and 100% in the MI271H group (*n* = 7). No significant differences in survival rates were found between the individual therapy groups.

### 3.2. Retention of Human Stem Cells in the Infarcted Heart 

To draw conclusions on the reliability of the intramyocardial injection method and cell retention, we investigated the numbers of human cells in the infarcted heart. Thereby, real time PCR was used for quantification of resident human cells in the murine tissue. Total RNA was isolated from the collected interlayers of cryosectioned hearts and Δ*CT*_mean_ of human GAPDH was quantitatively analyzed. In order to find out whether small amounts of transplanted cells can be detected in the donor, we initially verified the sensitivity of the methodology. To establish a calibration curve, we mixed murine heart tissue with different amounts of human BM stem cells. Relying on this we were able to detect at least 1000 cells (Figure 1B). Treatment with a five-fold CD133^+^ stem cell dose did not change the retention compared to 100,000 cells three weeks after MI. Furthermore, we only detected significantly lower cell retention in animals treated with 100,000 CD271^+^ MSC [24] (Figure 1C). 

### 3.3. Hemodynamic Functions

The cardiac performance was analyzed by hemodynamic measurement three weeks after cell transplantation. Table 1 shows significant improvements in the left ventricular ejection fraction (LVEF) and the velocity of pressure rise (dPmax, dPmin) both under baseline conditions and under stress using Dobutamine compared to untreated infarction [24]. However, we could not observe any therapeutic influence of a high-dose application in comparison to application of 100,000 cells. 

### 3.4. Cardiac Remodeling 

**Figure 2:**
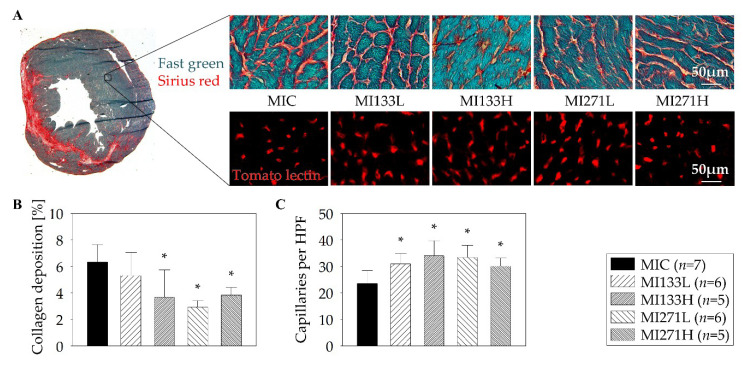
Cardiac remodeling three weeks post-myocardial infarction (MI). Histologically stained cross section of the infarcted heart as well as high-power fields (HPF) were performed to demonstrate degree of the fibrosis and capillary density at the remote area (**A**). No significant differences between the dose-dependent stem cell treatments with regard to fibrosis (**B**) and capillary density (**C**) were observed. Mean ± SD; * *p* ≤ 0.05 vs. MIC (Mann–Whitney *U* test).

Ligation of the LAD consistently resulted in a transmural MI with its typical histologic changes including the thinning of the left ventricular free wall (Fast green) and extensive collagen deposition (Sirius red) as well as a decrease in capillary density three weeks post-infarction (Figure 2A). Myocardial scar formation was tested for collagen deposition and evaluated by planimetry measurement. The reduced development of the infarction size in case of stem cell application in comparison to the MIC group is not significant. To determine interstitial fibrosis, three weeks post-MI we therefore examined the zone furthest away from the infarct scar (remote area) [27]. There is a significant difference in degree of fibrosis after stem cell treatment in contrast to MIC (Figure 2B). Three weeks after myocardial ischemia/reperfusion, hearts perfused with biotinylated tomato lectin were explanted following anti-Biotin staining of the heart cryosections. Capillary density was assessed by counting the number of capillaries. Thereby, all stem cell groups revealed significantly increased capillary density in the remote area in comparison with MIC (Figure 2C). However, no significant differences of fibrosis as well as capillary density between any of the stem cell treated groups were found. 

## 4. Discussion

Autologous HSC and MSC are available in very limited numbers. The question of a dose-response relationship therefore plays a central role in the clinical application of these stem cell populations. In this work, we show for the first time that a five-times higher dosage offers no significant increase with regard to the cardiovascular regenerative potential after MI. 

Small animal models can help to understand local effects but are not necessarily fully transferable to humans. In addition, animal experiments must promise a high prospect of success given their ethical burden. For this reason, we used cell numbers, which are typically transplanted intramyocardially in mice [28,29]. Obviously, it remains to be investigated which minimum amount in clinical use is sufficient for a beneficial effect in humans and to what extent further increases in dosage will influence the therapeutic outcome. In the clinical phase III study PERFECT, in which the safety and effectiveness of intramyocardially transplanted CD133^+^ HSC were examined within the framework of coronary artery bypass graft, cell suspensions with 0.5 to 5 × 10^6^ cells were used [30]. The knowledge gained here coincides with a meta-analysis of clinical data, where no differences in the effectiveness of the cardiovascular regeneration potential of transplanted stem cells at varying dosages (2 × 10^6^ − 60 × 10^6^) could be detected [31]. However, there are preclinical and clinical studies that indicate an increased effectiveness when using at least 1 × 10^6^ [32,33] or 5 × 10^5^ [34] cells. Yet in contrast, two clinical studies showed that the use of more than 2 × 10^7^ cultivated MSC can have even a negative influence on the therapeutic effect [35,36]. An increased inflammatory reaction or limited availability of nutrients in the ischemic tissue was discussed as a potential cause for this [36]. Such discrepant results with regard to the optimal cell dosage presumably occur due to the large number of different influencing factors in the different studies. These include the used experimental model, study design, cell types, purity levels and the form as well as the timing of the application. Moreover, pharmacokinetic and pharmacodynamic findings can be obtained separately for different cell types, indications and application forms. 

Fibrosis resulted in extensive collagen deposition and increased distance between myocytes three weeks after infarction. The chronic overload of the heart muscle after an infarction leads to massive collagen storage and matrix production with replacement of muscle cells by connective tissue. Fibrosis plays a central role in the deterioration of diastolic function, whereby a certain degree of fibrosis protects against ventricular deformation and ensures the transmission of force from the muscle cells to the entire ventricle. First, fibrosis only occurs perivascularly, then spreads to the interstitium between the muscle cells [27].

CD133^+^, as well as CD271^+^ populations, show a regenerative effect in the murine heart post-MI, as made clear from the enhanced hemodynamic functions and reduced remodeling. An increase in the transplanted cell dose led to a respective increase in cell survival only for CD271^+^ cells. The evidence that both cell types had a beneficial effect already at low doses which could not be further increased at high doses suggests one or several possibly interchangeable mechanisms of action [24] which may be successively overcompensated by harmful accumulation of cells. The hemodynamic function, as well as remodeling parameters, did not imply any therapeutic influence of a high-dose application in contrast to application of 100,000 cells. 

The adherence selection reveals the most common and characterized methodical tool to obtain BM-derived MSC applied in clinical trials. However, variations in culture conditions have heavily led to varying findings regarding MSC behavior and features in vitro [21,22]. Moreover, besides the phenotypical heterogeneity, this selection method is time-consuming and requires particle-free manufacturing. Although such MSC, selected by adherence, found a common application in allogenic therapies [37], there is no doubt that the inconsistent findings at the basic research level and differential stem cell-based clinical study outcomes require an alternative technique in order to obtain MSC. 

We detected CD271^+^ subpopulations in adult MSC [24,26]. Similar to us, others have reported that all fibroblast colony-forming cells (CFU-F) in human BM are present and rare [38,39]. While the total number of the MACS-purified CD271^+^ from sternal BM accounted approx. ~5500 per mL of the aspirate [26] in our study, the iliac crest BM aspirate accounted in 2–2.5 fold more MSC progenitors ranging from 1000–15,000 per mL of aspirate [38,40]. Hence, those studies have evidenced a linear relationship between numbers of mononuclear cells and mesenchymal progenitor cells as well as positive correlation between these mesenchymal progenitors and CFU-F counts. Recently, Ganguly and coworkers performed a study using iliac crest BM, showing an age-related decline in the numbers of the MSC [39]. Of note, a previous study has shown that even a dose of 50,000 uncultured un-concentrated MSC was efficacious following injection into a non-union fracture site [41]. In terms of autologous applications, it is the dose and volumetric concentration of MSC, rather than the purity, which appears to be critical. 

MSC’s immunosuppressive activity is one of the most fascinating issues in the field of adult stem cell research. In view of the broad spectrum of the allogeneic applications, the discovery of the underlying mechanisms is of crucial importance for their therapeutic use in the clinic. The quality of the examination of the cell cultures for any alterations in karyotypes before approval has now reached a level of clinical reliability and their implementation is of the utmost importance. However, Galleu and colleagues refer to allogeneic transplantation and discuss its complete unpredictability in terms of a therapeutic benefit, which raises concerns about the effectiveness of MSC. In this regard, they propose a concept—that patients should be stratified for MSC treatment according to their ability to kill MSC or that all patients could be treated with ex vivo apoptotic MSC [42]. In contrast, using transplants of freshly isolated, autologous BM stem cells, this would not be an expected issue. 

Overall, we conclude that the therapeutic benefit of BM-derived stem cell transplantation post-MI is largely dose-independent, and moreover, that potentially harmful amplification steps of the stem cells in culture can be omitted. 

## Figures and Tables

**Table 1:** Hemodynamic characteristics three weeks post-MI. Mean ± SD; * *p* ≤ 0.05 vs. MIC (Mann–Whitney *U* test; LVEDP—Left ventricular end diastolic pressure).

**Baseline** **Condition**	**SH** (*n* = 7)	**MIC** (*n* = 7)	**MI133H** (*n* = 8)	**MI133L** (*n* = 7)	**MI271H** (*n* = 7)	**MI271L** (*n* = 7)
**dPmax** [mmHg/s]	4159.10* ± 325.24	2613.64 ± 372.44	2809.09 ± 126.14	3869.36 ± 688.17	3510.26 ± 552.77	3868.91* ± 290.33
**LVEF** [%]	55.60* ± 5.44	25.81 ± 2.76	37.53 ± 4.37	41.11* ± 4.73	40.01 ± 3.91	45.66* ± 2.04
**dPmin** [mmHg)/s]	−3835.77* ± 242.96	−2227.65 ± 395.24	−2352.85 ± 129.00	−3353.57 ± 650.47	−3241.84 ± 548.76	−3222.29 ± 269.63
**τ** (Tau) [ms]	7.40 ± 1.14	7.72 ± 1.85	9.44 ± 2.65	8.00 ± 3.02	11.63 ± 4.45	7.59 ± 1.58
**LVEDP** [mmHg]	3.10 ± 0.85	2.35 ± 0.77	2.72 ± 2.96	2.54 ± 1.09	4.69 ± 2.22	2.60 ± 0.45
**Stress** **condition**	**SH** (*n* = 7)	**MIC** (*n* = 7)	**MI133H** (*n* = 8)	**MI133L** (*n* = 7)	**MI271H** (*n* = 7)	**MI271L** (*n* = 7)
**dPmax** [mmHg/s]	12794.03* ± 813.35	7776.74 ± 1209.54	7152.38 ± 1102.03	9588.91 ± 1417.98	9489.74 ± 920.56	9399.51 ± 873.92
**LVEF** [%]	85.63* ± 4.46	46.86 ± 8.20	58.53 ± 9.03	57.23 ± 5.21	74.37 ± 6.00	65.57 ± 5.91
**dPmin** [mmHg/s]	−11828.26* ± 1948.01	−6372.09 ± 1349.94	−5400.93 ± 845.98	−7214.74 ± 1031.34	−6499.11 ± 752.13	−5975.43 ± 555.27
**τ** (Tau) [ms]	4.11 ± 0.41	6.14 ± 1.32	6.88 ± 2.00	6.03 ± 2.17	5.66 ± 2.46	3.43 ± 0.24
**LVEDP** [mmHg]	2.51 ± 0.26	5.71 ± 2.80	4.90 ± 4.47	2.69 ± 1.51	3.66 ± 2.09	2.09 ± 0.28
